# Personality disorders in substance abusers: Validation of the DIP-Q through principal components factor analysis and canonical correlation analysis

**DOI:** 10.1186/1471-244X-5-24

**Published:** 2005-05-24

**Authors:** Morten Hesse

**Affiliations:** 1 Aarhus University, Centre for Alcohol and Drug Research, Kobmagergade 26E, 1150 Copenhagen C, Denmark

## Abstract

**Background:**

Personality disorders are common in substance abusers. Self-report questionnaires that can aid in the assessment of personality disorders are commonly used in assessment, but are rarely validated.

**Methods:**

The Danish DIP-Q as a measure of co-morbid personality disorders in substance abusers was validated through principal components factor analysis and canonical correlation analysis. A 4 components structure was constructed based on 238 protocols, representing antagonism, neuroticism, introversion and conscientiousness. The structure was compared with (a) a 4-factor solution from the DIP-Q in a sample of Swedish drug and alcohol abusers (N = 133), and (b) a consensus 4-components solution based on a meta-analysis of published correlation matrices of dimensional personality disorder scales.

**Results:**

It was found that the 4-factor model of personality was congruent across the Danish and Swedish samples, and showed good congruence with the consensus model. A canonical correlation analysis was conducted on a subset of the Danish sample with staff ratings of pathology. Three factors that correlated highly between the two variable sets were found. These variables were highly similar to the three first factors from the principal components analysis, antagonism, neuroticism and introversion.

**Conclusion:**

The findings support the validity of the DIP-Q as a measure of DSM-IV personality disorders in substance abusers.

## Background

The dimensional structure of personality disorders is a growing area of research. Several studies have presented factor models over the past decade [1-4]. Advanced statistical methods have been applied, but a serious limitation is that it is impossible to disentangle the effects of the instrument used, sample characteristics, and measurement error. Any given study may find a particular factor structure, but will be unable to determine whether this factor structure is a reflection of the instrument's characteristics, the sample's particular characteristics, random error, or a general factor structure underlying personality disorders. These same limitations have applied to studies that have attempted to link personality disorders with models of normal personality [5,6].

In order to overcome these limitations, O'Connor [7] conducted a meta-analysis of personality disorders in order to produce a consensual factor structure based on different measures. He included 33 different published correlation matrixes of personality disorders in his analysis, using a wide array of measurement instruments and samples. He produced aggregated factors to produce what he called a consensus structure of personality disorders. He reported both 2, 3 and 4-component consensus solutions.

The 2-component solution appeared to represent an internalizing/externalizing dimension, the 3-component solution represented low agreeableness, low extroversion and neuroticism, whereas the 4-component solution represented these three plus conscientiousness (represented only by obsessive-compulsive personality disorder). In the following, I shall refer to the low agreeableness factor as antagonism, following Blackburn [[8], p. 61], and to the low extroversion factor as introversion for simplicity.

Substance abusers are a good target for the study of personality disorders, because of the high comorbidity of personality disorders and substance abuse [9].

The focus of the present study is validation of the DSM-IV section of the Danish translation of the DSM-IV and ICD-10 Questionnaire [10]. Validation in this case requires that its factor structure is identical to the factor structure of the Swedish original, and identical, or at least highly similar, to the factor structure of personality disorders in general. A secondary purpose of the study is to assess the latent dimensions underlying the relationship between self-reported traits representing personality disorder and observer reported traits representing the same personality disorders.

## Method

### Participants

A sample of clients from several Danish substance abuse treatment settings was included, with both inpatient and outpatient settings, rural settings and urban settings, methadone and none-methadone settings.

A total of 238 different substance abusers (hereafter referred to as "clients") in treatment were administered the DIP-Q and rated by staff members. Of the clients, 28% were women, 68% were men, and for 4%, gender was not reported. The mean age of clients was 33.4 years (standard deviation [SD] = 9.4). Of the clients, 50% were from inpatient settings for substance abuse treatment, and 50% were from outpatient treatment settings for substance abuse.

Each client was rated by a mean of 2.0 staff members (SD = 1.8). Clients who had only been seen in individual counseling or psychotherapy constituted 37% of the sample, 8% was rated only by staff members who had not provided individual counseling to the client, and the remaining 55% had been rated by staff members who had seen the client in a variety of settings, including both individual counseling and group settings.

A total of 59 raters participated, 17 men and 40 women. Most ratings took place at face-to-face meetings with the author, but 36 clients were rated on forms, and subsequently mailed to the author by letter or conducted as telephone interviews.

Most patients from inpatient settings (60%) and 17% from outpatient settings were rated by more than one staff members. In the canonical correlation analysis, the mean of the different raters' ratings was used.

The Swedish sample consisted of follow-up samples from two different treatment institutions (N = 133). One sample consisted of 60 substance abusers admitted for treatment around 1989 who were assessed at a 15 year follow-up in 2004, whose mean age in 2004 was 44.5 years (range:35 – 66). The second sample was from a five-year follow-up of 54 alcohol abusers from a private inpatient treatment centre in Sweden. Gender and age was not available for this subsample. The remaining 19 subjects were adult males from a project for criminal youth (mean age: 30, range: 21–45).

### Instruments

#### The DIP-Q

The DSM-IV and ICD-10 Personality Questionnaire [DIP-Q] was used as the measure of personality pathology. The DIP-Q is a self-report questionnaire for screening for DSM-IV and ICD-10 PDs, plus schizotypal disorder in ICD-10 [10,11]. The instrument is highly similar to other questionnaires measuring PDs, such as the SCID-IIQ, and the PDQ-R. It consists of 151 statements that must be rated as true or false and three self-rating scales: severity of current events, global assessment of functioning, axis V of the DSM-IV for past year and global assessment of functioning for recent weeks. The DIP-Q was constructed through a consensus process. First, four psychiatrists worked together select a range of statements considered representative of the diagnostic criteria for each personality disorder. These statements could answered in a true/false format. The representative statements were then reviewed and validated by a second set of independent psychiatrists [12]. A translation and an English version was made available from the Swedish authors. No details of this translation were available. Therefore, a new Danish translation was made based on the English and Swedish versions, and then compared with the original translation. Any discrepancies in the items were checked against the English and Swedish versions, and revised if necessary.

Studies show indications of concurrent [11] and predictive [13] validity of the instrument. Responses are used to determine the presence of criteria for each PD. In the present study criteria counts are used as measures of personality disorder features. Clients (N = 238) filled in the DIP-Q typically after at least 1 week of treatment. The DIP-Q questionnaires were sent to the Centre for Alcohol and Drug Research, where they were scored by the author.

#### Staff ratings

A subset of the sample (N = 152) were also rated by staff members involved in their treatment. Rating scales were used for staff ratings, rating the degree of personality pathology on each of the 10 PDs in the DSM-IV on a scale from 0 to 100. Each PD was assessed with only one scale. Three keywords were added for each disorder as prompts (e.g.: Paranoid: Guarded, jealous, expecting malevolence). Inter-rater agreement for this instrument has been reported [14]. Raters rated the patients at staff meetings with the author present, or filled in the rating scales on their own and mailed them to the author. Raters were not shown the results of the questionnaire before they had completed their rating of the client. Thus, raters were blind to the results of the questionnaire.

### Statistical analyses

Following O'Connor [7], principal components analysis was conducted for 4 components, and Varimax rotations were performed. The coefficient of congruence (congruence) was calculated for the entire factor solutions with both O'Connor's consensus model and between the Danish and Swedish samples. The congruence is a commonly used statistic in factor analysis to test replicability of components, and it indexes the proportional similarity between two sets of loadings, and yields values that are essentially identical to the values produced by other indices. Standard interpretation of the congruence coefficient suggests that 0.90 indicates acceptable fit. In the O'Connor meta-analysis, the range of congruence with consensus solution for each individual dataset went as low as 0.60, but the mean congruence was around 0.80 for individual datasets with the consensus solution. This, however, included instruments that were based on quite different conceptualizations of personality disorders, such as the first version of the MCMI, and some very small datasets. Based on these reports, it was decided that an congruence of 0.80 or higher was an acceptable replication of the factor structure for the present report. However, a congruence of 0.90 or higher is desirable. The same factor analysis was conducted on the staff ratings, and congruence between the solution found for the staff ratings and the DIP-Q, and between the solution found for the staff rating and the consensus model were calculated.

However, beyond the factor structure, an equally important point is to what degree the same latent variables link the self-reported personality disorder traits and the staff reported severity of personality disorder. A canonical correlation analysis was conducted to assess the latent dimensions connecting self-reported personality disorder on the DIP-Q and staff reported personality disorder severity [15]. Canonical correlation analysis is an approach that assesses the overall relationships between 2 sets of variables. It is similar to factor analysis, in that it identifies synthetic variables (or latent dimensions) underlying observed variables, but does so by producing maximally correlated variables across to sets of variables. The canonical correlation analysis produces several statistics for the relationships between synthetic variables, and between observed and latent or synthetic variables: the *canonical correlation coefficient *represents the Pearson r relationship between the two synthetic variables on a given canonical function. A canonical function is a set of standardized canonical function coefficients (from two linear equations) for the observed predictor and criterion variable sets. This function is analogous to the components in a principal components analysis, in that it represents a dimension in the data that is uncorrelated with other dimensions. *Standardized canonical function coefficients *are the standardized coefficients, and are similar to beta coefficients in regression analysis. A *structure coefficient *(r_s_) is the correlation between an observed variable and a synthetic variable.

The analyses planned were as follows:

1. A 4-component factor analysis was constructed for the Swedish and Danish samples of drug abusers.

2. The congruence scores across samples of each of the components and the whole models were analyzed. First, each pair of components were compared, and then the pair with the maximum congruence was selected. Based on this, components received labels.

3. The congruence of the loadings between the Danish sample and the consensus model were calculated, for both the whole model and each of the factors.

4. A 4-component factor analysis was constructed for the staff ratings on the subset of the Danish sample of substance abusers who were rated by staff members.

5. Canonical correlation analysis was used to assess the latent dimensions linking staff members' reports with DIP-Q scales. The canonical correlation analysis was conducted along the guidelines of Sherry and Henson [15].

## Results

### Factor analysis

The factor loadings as well as congruence statistics from the factor analysis are summarized in table 1. Readers are referred to O'Connor for the factor loadings from the consensus model [7].

**Table 1:** Factor loadings for the Danish DIP-Q and congruence scores

	**Antagonism**	**Introversion**	**Neuroticism**	**Conscientiousness**	**h^2^**
Paranoid	**0.46**	**0.51**	0.24	0.06	0.53
Schizoid	-0.07	**0.86**	0.09	0.10	0.76
Schizotypal	**0.49**	**0.63**	0.07	0.13	0.66
Antisocial	**0.81**	0.10	-0.04	-0.13	0.69
Borderline	**0.70**	0.29	0.39	0.07	0.73
Histrionic	**0.74**	-0.08	0.20	0.10	0.60
Narcissistic	**0.67**	0.17	0.01	0.40	0.65
Avoidant	0.05	0.44	**0.74**	0.18	0.78
Dependent	0.18	-0.02	**0.91**	0.04	0.86
Obsessive-compulsive	0.07	0.14	0.14	**0.93**	0.91

**Congruence coefficients**					

With Swedish sample	0.93	0.96	0.95	0.89	

With Consensus model	0.96	0.89	0.93	0.90	

**Notes: **Loadings greater than |.45| are in boldface.

The four-components solution showed good congruence between the Danish and the Swedish version (0.93), and good congruence between the Danish DIP-Q and the consensus solution (0.92).

The congruence of the individual factors were mostly above the 0.90 cut-off. Two exceptions were noted: the conscientiousness factor was just under adequate congruence between the two samples, and the introversion factor was just below cut-off in similarity between the Danish DIP-Q and the consensus model. The lack of congruence between the DIP-Q introversion and the consensus sample was due mainly to the low negative loading of histrionic personality disorder on introversion. In the consensus model, histrionic PD had a high negative loading on introversion (-0.53).

The factor structure for the staff ratings is presented in table 2. Congruence with neither the DIP-Q nor the consensus model was as high as that found between the two versions of the DIP-Q, or between DIP-Q and the consensus model. The antagonism and conscientiousness components were both congruent with the predicted models, but the neuroticism and introversion factors had much lower congruence scores. The main differences were that the narcissism scale had a high negative loading on the neuroticism component, and that avoidant personality disorder had a lower loading on introversion, while antisocial had an unexpected loading on the introversion component.

**Table 2:** Factor loadings for the staff ratings and congruence scores

	**Antagonism**	**Neuroticism**	**Introversion**	**Conscientiousness**	**h^2^**
Paranoid	**0.46**	0.40	**0.49**	0.30	0.70
Schizoid	-0.03	0.36	**0.76**	0.14	0.73
Schizotypal	0.21	0.02	**0.83**	0.08	0.73
Antisocial	**0.67**	-0.13	0.44	0.01	0.67
Borderline	**0.81**	0.30	-0.03	-0.01	0.75
Histrionic	**0.72**	-0.12	0.07	0.13	0.55
Narcissistic	**0.56**	**-0.47**	0.28	0.27	0.69
Avoidant	-0.30	**0.76**	0.26	0.10	0.75
Dependent	0.22	**0.81**	0.12	-0.06	0.73
Obsessive-compulsive	0.12	0.00	0.16	**0.96**	0.96

Congruence coefficients					
With DIP-Q	0.95	0.85	0.89	0.91	

With consensus model	0.92	0.84	0.78	0.92	

**Notes: **Loadings greater than |.45| are in boldface.

### Canonical correlation analysis

Canonical correlation analysis was conducted to assess the dimensions that underlie the relationship between the DIP-Q and the staff ratings.

The correlations between the individual DIP-Q scales and staff members' ratings of patients are summarized in table 3. The correlations are quite modest in size, which is typical of associations between ratings based on clinical impression and self-report [16,17], and between self-report and observations from other informants [18].

**Table 3:** Canonical Solution for staff ratings predicting DIP-Q profiles

		Function 1 (agreeableness)			Function 2 (neuroticism)			Function 3 (extroversion)			h^2^
Variable	Conv r	Coef	r_s_	r_s_^2 ^(%)	Coef	r_s_	r_s_^2 ^(%)	Coef	r_s_	r_s_^2 ^(%)	

**DIP-Q**											
Paranoid	0.15	-0.01	-0.23	5	0.05	0.39	15	0.23	-0.03	0	0.21
Schizoid	0.21	0.06	0.11	1	-0.11	0.19	4	-0.91	**-0.82**	67	**0.72**
Schizotypal	0.19	0.29	-0.14	2	0.19	0.35	2	0.12	-0.23	5	0.20
Antisocial	0.49	-0.65	**-0.87**	76	0.08	0.21	4	0.04	-0.06	0	**0.80**
Borderline	0.44	-0.42	**-0.57**	32	0.29	**0.57**	32	-0.25	-0.12	1	**0.66**
Histrionic	0.21	-0.13	-0.42	18	-0.36	0.06	0	0.25	0.22	5	0.23
Narcissistic	0.22	-0.23	**-0.49**	24	-0.02	0.10	1	-0.29	-0.15	2	0.27
Avoidant	0.29	0.25	0.21	4	0.55	**0.76**	58	-0.17	-0.11	1	**0.63**
Dependent	0.27	0.15	0.08	1	0.46	**0.75**	56	0.45	0.30	9	**0.66**
Obsessive-compulsive	0.03	0.01	0.08	1	-0.40	-0.04	0	0.30	0.04	0	0.01

**R_c_**			0.61			0.55			0.47		

**Staff ratings**											
Paranoid		0.15	-0.35	12	0.67	**0.53**	28	0.15	-0.03	0	0.40
Schizoid		0.02	-0.11	1	-0.31	0.04	0	-0.58	**-0.54**	29	0.31
Schizotypal		0.03	-0.29	8	-0.16	-0.06	0	-0.55	**-0.46**	21	0.30
Antisocial		-0.69	**-0.85**	72	0.01	0.03	0	0.18	0.06	0	**0.73**
Borderline		-0.48	**-0.70**	49	0.32	0.40	16	-0.18	0.03	0	**0.65**
Histrionic		0.19	-0.35	12	-0.13	-0.15	2	0.51	0.40	16	0.31
Narcissistic		-0.30	**-0.65**	42	-0.35	-0.43	18	-0.22	0.01	0	**0.61**
Avoidant		0.08	0.32	10	0.30	**0.53**	28	-0.16	-0.32	10	**0.49**
Dependent		0.03	-0.03	0	0.22	**0.62**	38	0.26	0.00	0	0.39
Obsessive-compulsive		0.11	-0.08	1	-0.23	-0.23	5	0.54	0.33	11	0.17

**Notes. ***Conv. r: *Convergent correlation coefficient (simple bivariate correlation). *Coef *= standardized canonical function coefficient; *r*_s _= structure coefficient; *r*_s_^2 ^(%) = squared structure coefficient; *h^2^*= communality coefficient. *R_c _*is the canonical correlation coefficient. Structure coefficients (r_s_) greater than |.45| are in boldface. Communality coefficients (h^2^) greater than |.45| are in boldface.

A canonical correlation analysis was conducted using the 10 rating scales as predictors and the 10 DIP-Q scales as dependent variables. The analysis yielded 10 functions with squared canonical correlations (r_c_^2^) ranging from 0.38 to less than 0.001 for each successive function. These 10 functions will be referred to as functions 1–10 in the following.

The full model across all functions was statistically significant using the Wilks's criterion (λ = .21, F(100, 971.3) = 2.35, p < .001). Because Wilks's λ represents the variance unexplained by the model, 1 – λ yields the full model effect size in an r^2 ^metric. Thus, for the set of 10 canonical functions, the r^2 ^type effect size was .79, which indicates that the full model explained nearly all, about 79%, of the variance shared between the variable sets.

Significance testing of functions later than function 4 showed that these were not significant (F(36,608.8) = 1.26, p = 0.144). Only functions 1–3 explained more than 10% of the variance each (and 20% of remaining variance after the extraction of prior functions), and these are the only ones retained for further analysis.

Inspection of table 3 shows functions 1–3 are highly similar in content to the three first factors derived from the principal components analysis, and can be labelled agreeableness, neuroticism and extroversion, respectively.

The agreeableness function yielded a strong correlation between staff ratings and DIP-Q (R_c _= 0.61). The structure coefficients of the agreeableness factor are high for antisocial, borderline and narcissistic personality disorder in both the DIP-Q and the staff ratings (all>0.45). The standardized coefficient was quite small for narcissistic personality disorder, indicating that this variable is somewhat redundant in this context.

The canonical correlation coefficient for the **neuroticism **factor was 0.55. In the neuroticism function, the dependent and avoidant personality disorder had strong structure coefficients with both methods, and borderline personality had a strong structure coefficient for the DIP-Q.

The canonical correlation coefficient for the **extroversion **factor was 0.47. This function had only one strong structure coefficient, schizoid personality disorder on the DIP-Q (coefficient = -0.82). Small positive coefficients were found for dependent and histrionic personality disorders, and in the staff reported version, schizoid and schizotypal personality disorder have strong negative loadings, a small negative loading was seen for avoidant personality disorder, and small positive loadings were found for histrionic and obsessive-compulsive personality disorder.

## Discussion

The present study showed that the factor structure of the DIP-Q is invariant over the Danish and Swedish version for samples of drug abusers. It has adequate congruence with a consensus factor structure derived from a meta-analysis of several different instruments.

It was also found that a similar set of latent functions explain the relationship between the DIP-Q scales and staff reported personality pathology, although the observed correlations between the personality disorder scales and the staff ratings were small. These associations are important, because they link the factor structure observed to external and independent ratings of the subjects' pathology.

The factor structure of staff reported personality pathology was not as close to the consensus model as was the factor structure of the DIP-Q. The factor structure of staff reported pathology was only a close match on antagonism and conscientiousness. Staff reported neuroticism had a strong negative loadings on narcissism (curiously, similarly to the MCMI's high correlation between narcissism and neuroticism [19]. However, the congruence for neuroticism and introversion was still close to the mean of the values for congruence with varimax rotation reported in O'Connor's meta-analysis [[7], table 4].

### Similarity and differences between the canonical solution and the factor analysis solutions

Overall, the factor loadings and the canonical correlations are quite similar. The most striking difference is, of course, that the conscientiousness factor did not emerge in the canonical correlation analysis. However, obsessive-compulsive personality disorder, the only disorder with a strong loading on this factor in the presented factor analyses and in O'Connor's meta-analysis, had only very weak inter-rater agreement [14], thus making it very hard for the two datasets to converge on this dimension. Also, this factor is statistically weak, because the latent dimension, maladaptive conscientiousness, is covered by only a single personality disorder in the DSM-IV system. It is, however, conceptually important.

Generally, the principal components analysis extracted most of the variance in all 10 personality disorders, as is evident in the high communalities in table 1 and 2, whereas much variance was unaccounted for in the canonical correlation analysis, as reflected in the low communalities in table 3. This overall difference may reflect overall significant and possibly systematic differences between what people observe themselves and what others see in them [18]. In other words, much of what we experience may not be reflected in what others see, and much of what others see may not be reflected in how we describe ourselves. This may derive from lack of insight into one's behaviour: a severely narcissistic person may experience himself as healthy and extroverted; it may also stem from the difficulties inherent in inferring motives from overt behaviour: e.g., a person with schizoid personality disorder and a person with avoidant personality disorder may both appear withdrawn and become uncomfortable with close contact, but their inner world and perception of other people may differ substantially.

### Limitations

The present study is limited to drug and alcohol abusers, and is limited by being based on convenience samples. However, previous research has not shown meaningful differences in the factor structure of personality measures, including personality disorder measures, between various types of samples [20]. Substance abusers have the advantage that all personality disorders are more common among substance abusers than in the general population, leading to more variance in such a sample [9]. Another limitation is sample size. With a larger sample, approximately 1500, factor analysis could be done on the items of the DIP-Q rather than the composite scales. However, while this is a limitation, it does allow comparison of the present analysis with the many analyses that have been conducted on personality disorders in a similar manner.

## Conclusion

The Danish DIP-Q has a latent factor structure in drug and alcohol abusers that is consistent with the original Swedish, and similar to the general factor structure in personality disorders. The latent structure is valid, in as far as it converges with staff members' impression of the personality disorders of respondents.

## Abbreviations

DIP-Q: The DSM-IV and ICD-10 Personality Questionnaire

PD: Personality Disorder

SCID-II: The Structured Clinical Interview for the DSM, Axis II section

## Competing interests

The author(s) declare that they have no competing interests.

## Authors' contributions

MH planned, designed and conducted the study, and wrote the manuscript.

## Pre-publication history

The pre-publication history for this paper can be accessed here:


